# Combination of immunohistochemistry, FISH and RT-PCR shows high incidence of Xp11 translocation RCC: comparison of three different diagnostic methods

**DOI:** 10.18632/oncotarget.16481

**Published:** 2017-03-22

**Authors:** Hyun Jung Lee, Dong Hoon Shin, Gyu You Noh, Young Keum Kim, Ahrong Kim, Nari Shin, Jung Hee Lee, Kyung Un Choi, Jee Yeon Kim, Chang Hun Lee, Mee Young Sol, Seo Hee Rha, Sung Woo Park

**Affiliations:** ^1^ Department of Pathology, School of Medicine, Pusan National University, Yangsan, Korea; ^2^ Department of Urology, School of Medicine, Pusan National University, Yangsan, Korea; ^3^ Research Institute for Convergence of Biomedical Science and Technology, Pusan National University Yangsan Hospital, Yang San, Korea; ^4^ Department of Pathology, Donga University Hospital, Busan, Korea

**Keywords:** TFE3, RT-PCR, FISH, renal cell carcinoma, FFPE, Pathology Section

## Abstract

We evaluated the frequency of translocation renal cell carcinoma (RCC) by reverse transcription polymerase chain reaction (RT-PCR) and how well the TFE3 immunoreactivity is concordant with *TFE3* gene translocation status proved by fluorescence in situ hybridization (FISH) assay and RT-PCR. TFE3 and Cathepsin K expression was analyzed by immunohistochemistry in 185 RCC cases, and 48 cases either of more than weak expression of TFE3 or of positivity for Cathepsin K were done for FISH analysis and RT-PCR. All the RT-PCR positive cases were confirmed by cloning and sequencing. Of the 14 cases with strong nuclear TFE3 expression, 12 showed a break-apart signal by FISH. *ASPL-* and *PRCC*-*TFE3* translocations were detected in 13 and one case, respectively, by RT-PCR. Of 21 cases with weak TFE3 expression, five were translocation-positive by FISH. *ASPL-*, *PRCC-*, and *PSF*-*TFE3* translocations were detected by RT-PCR (*n*=3, 3, and 1, respectively). All 13 TFE3-negative/cathepsin K-positive cases were negative by FISH and two each harbored *ASPL-* and *PRCC*-*TFE3* translocations that were detected by RT-PCR. A high rate of TFE3 immunoreactivity (8.6%) was confirmed by RT-PCR (13.5%) and FISH (9.7%). Higher translocation rate of RT-PCR means RT-PCR detected translocation in TFE3 weak expression group and only cathepsin K positive group more specifically than FISH. Thus, RT-PCR would complement FISH analysis for detecting translocation RCC with fusion partners.

## INTRODUCTION

Xp11 translocation renal cell carcinoma (RCC) is characterized by chromosome translocations involving the *TFE3* gene at the Xp11 breakpoint [1, 2]. TFE3 is a member of the microphthalmia-associated family of basic helix-loop-helix leucine zipper transcription factors and is involved in transforming growth factor (TGF)-β-induced transcription during cell growth, and proliferation [3]. Fusion partners of *TFE3* include *PRCC*, *PSF (SFPQ1)*, *NonO*, *ASPL (ASPSCR1)*, *CLTC* and *DVL2* [4, 5] and some studies have indicated that translocation RCCs with different translocations show different morphologic features [6, 7]. Therapies targeting vascular endothelial growth factor receptor and mammalian target of rapamycin may benefit patients with Xp11 translocation RCC [8, 9]; the MET signaling pathway is another possible target, since it is activated by *ASPL*-*TFE3* fusion [10]. Although translocation RCC was initially described in children and has a relatively indolent course [3], recent studies have identified RCCs with *TFE3* rearrangement in adults have shown a more aggressive course [6, 11, 12]. Therefore, differentiating Xp11 translocation RCC from other subtypes is of clinical importance and more than of academic interest.

Xp11 translocation RCC is primarily identified by immunohistochemical detection of TFE3 protein and fluorescence *in situ* hybridization (FISH) detection of a break-apart signal. The former method is cheaper and more convenient but has questionable sensitivity and specificity, whereas the latter is regarded as the gold standard but is expensive and labor intensive [13–17]. Moreover, fusion partners cannot be easily distinguished by FISH. RNA sequencing is a recently developed tool for detecting translocation fusion partners [5], but is too costly for routine clinical use. Reverse transcription polymerase chain reaction (RT-PCR) provides a less expensive and simpler alternative, but RT-PCR using formalin fixed paraffin embedded (FFPE) tissue is not always successful due to suboptimal RNA quality.

To compare three methods for diagnosing Xp11 translocation RCC, the present study examined the results of TFE3 immunohistochemistry, FISH, and RT-PCR using FFPE tissue. The RT-PCR results were confirmed by cloning and sequencing. We also examined the expression of cathepsin K, which acts downstream of TFE3 and is overexpressed in Xp11 translocation RCC in order to identify additional cases.

## RESULTS

### Clinicopathologic characteristics of patients

**Figure 1:**
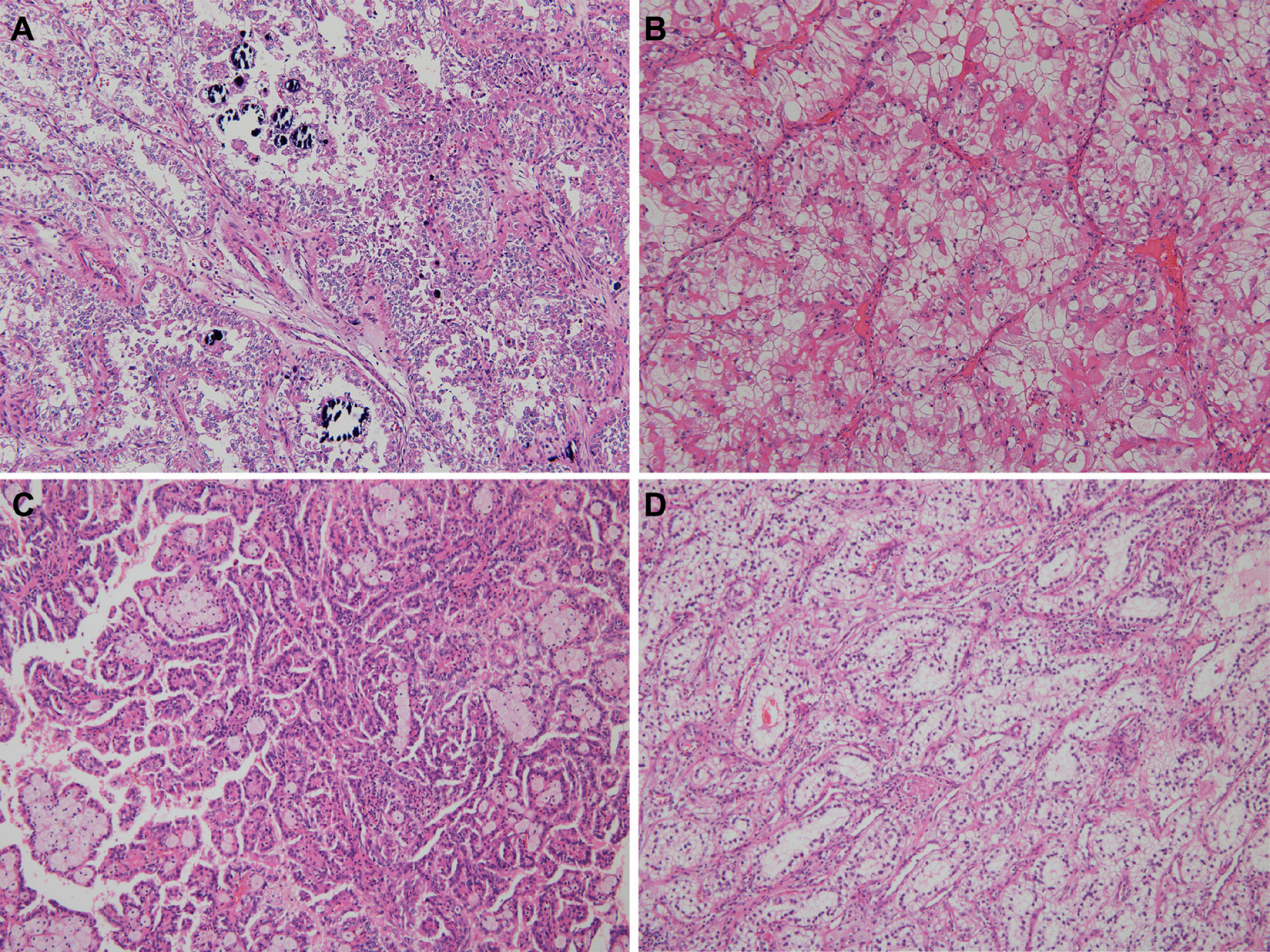
Morphologic features of translocation renal cell carcinoma, according to fusion partners **A**. The tumor showed psammoma bodies with nested alveolar pattern (*ASPL*-*TFE3* fusion). **B**. Voluminous expansile eosinophilic cytoplasm was seen in *ASPL*-*TFE3* translocation renal cell carcinoma. **C**. Papillary architecture with eosinophilic cytoplasm was seen in *PRCC*-*TFE3* translocation renal cell carcinoma. **D**. Clear cytoplasm with subnuclear vacuolation was identified in *PSF*-*TFE3* translocation renal cell carcinoma.

A total 185 study population consisted of 132 males and 53 females with a mean age of 52 years (range: 34-88 years). None of the patients had a history of malignancies. Patients were diagnosed with clear cell RCC (*n* = 153), papillary RCC (*n* = 20), chromophobe RCC (*n* = 10), and translocation RCC (*n* = 2). Clinicopathologic features along with FISH and RT-PCR results of 48 cases are shown in Table 2. The morphological features of translocation RCC are shown in Figure 1.

**Table 1:** Primer pairs used for RT-PCR

ASPL-TFE3 (FORW)	5′-AAAGAAGTCCAAGTCGGGCC-3′
ASPL-TFE3 (REV)	5′-CGTTTGATGTTGGGCAGCTC-3′
PRCC-TFE3 (FORW)	5′-GCCTCAATCTGCCCCCTCCAAT-3′
PRCC-TFE3 (REV)	5′-CGAGTGTGGTGGACAGGTACT-3′
CLTC-TFE3 (FORW)	5′-GTCGCGTTGTTGGAAAGTATTG-3′
CLTC-TFE3 (REV)	5′-AAAAGGGCCTTTGCCTCGGT-3′
PSF-TFE3 (FORW)	5′-TGGTGGTGGCATAGGTTATG-3′
PSF-TFE3 (REV)	5′-CGTTTGATGTTGGGCAGCTC-3′
NonO-TFE3 (FORW)	5′-GAGAAACTAGACACAGCA-3′
NonO-TFE3 (REV)	5′-TGTACACATCAAGCAGAC-3′
TFE3 wild 821-1008 (FORW)	5′-GGCAGCAGGTGAAACAGTAC-3′
TFE3 wild 821-1008 (REV)	5′-CTCTGAGCTGGACCCGATGGTGA-3′

**Table 2:** Clinicopathologic features and comparing FISH assay with RT-PCR

Case	Age	Sex	Location	Tumor Size (cm)	TNM	Grade	TFE3 IHC	CathepsinK IHC	TFE3 Wildtype 821-1008	TFE3 FISH	%TFE3 Split Signals	RT-PCR
1	76	F	Right	5.4	pT3aN0M0	3	+++	-	-	+	20%	ASPL-TFE3
2	79	M	Right	2.0	pT1aN0M0	4	+++	-	-	+	15%	ASPL-TFE3
3	67	F	Right	2.5	pT3aN1M0	3	+++	-	-	+	72%	ASPL-TFE3
4	36	F	Left	4.6	pT1bN0M0	3	+++	+++	-	+	25%	ASPL-TFE3
5	71	F	Left	10.0	pT1aN0M0	3	+++	+++	-	+	16%	ASPL-TFE3
6	34	F	Right	8.5	pT2aN0M0	2	+++	++	-	+	25%	ASPL-TFE3
7	80	M	Right	4.9	pT3aN0M0	2	+++	+++	-	+	16%	ASPL-TFE3
8	75	F	Left	10.5	pT3aN0M0	3	++	-	-	+	17%	ASPL-TFE3
9	57	F	Right	5.5	pT3aN0M1	3	+++	-	-	+	83%	PRCC-TFE3
10	78	M	Left	4.6	pT1bN1M1	3	++	-	-	+	88%	ASPL-TFE3
11	72	F	Left	3.5	pT1aN0M0	3	++	-	-	-	7%	ASPL-TFE3
12	62	F	Right	3.8	pT3aN0M1	3	++	-	-	-	5%	ASPL-TFE3
13	67	M	Right	3.2	pT1aN0M1	3	++	-	-	+	85%	ASPL-TFE3
14	78	M	Left	7.2	pT3aN1M1	4	+++	++	-	+	15%	ASPL-TFE3
15	69	M	Right	5.1	pT1bN0M0	3	+	-	-	-	2%	ASPL-TFE3
16	45	M	Left	9.5	pT3aN0M0	3	+	-	-	+	20%	
17	75	M	Right	4.7	pT3aN0M0	3	+	-	-	-	2%	
18	78	F	Left	2.4	pT1aN0M0	2	+	+++	+	+	65%	PSF-TFE3
19	83	M	Right	4.0	pT1aN0M0	2	+	-	-	-	5%	
20	58	M	Left	9.0	pT2aN0M0	2	+	-	+	-	4%	
21	72	M	Left	6.0	pT3aN0M0	3	+	-	+	-	2%	PRCC-TFE3
22	63	F	Left	6.4	pT1bN0M0	3	+	-	-	+	37%	ASPL-TFE3
23	46	M	Left	3.5	pT1aN0M0	2	+	-	-	-	5%	PRCC-TFE3
24	37	M	Right	4.0	pT1aN0M0	2	+	-	+	-	2%	PRCC-TFE3
25	61	M	Right	2.5	pT1aN0M0	2	+	-	+	-	1%	
26	76	M	Left	4.8	pT1bN0M0	2	+	-	-	-	0%	
27	77	M	Right	10.4	pT2bN0M0	2	+	-	-	-	0%	
28	56	M	Left	3.5	pT1aN0M0	2	+	-	-	-	0%	
29	77	M	Left	2.8	pT1aN0M0	2	+	-	+	-	1%	
30	62	M	Right	2.4	pT1aN0M0	2	+	-	-	-	0%	
31	61	M	Left	2.0	pT1aN0M0	2	+	++	-	+	67%	ASPL-TFE3
32	69	M	Left	7.9	pT2aN0M0	2	+	-	+	-	2%	
33	41	M	Left	4.8	pT1bN0M0	3	+	-	-	+	16%	
34	70	M	Left	4.0	pT3aN1M0	4	+	-	-	-	0%	
35	56	M	Right	3.3	pT1aN0M0	2	+	-	+	-	5%	
36	57	M	Right	4.7	pT1bN0M0	2	-	++	-	-	3%	ASPL-TFE3
37	69	F	Left	2.1	pT1N0M0	2	-	++	-	-	2%	PRCC-TFE3
38	63	M	Right	4.1	pT1bN0M0	3	-	+++	-	-	2%	ASPL-TFE3
39	62	M	Right	2.5	pT1aN0M0	2	-	+++	-	-	5%	PRCC-TFE3
40	42	M	Left	4.0	pT1N0M0	3	-	+++	-	-	3%	
41	76	M	Right	5.0	pT3aN0M1	2	-	+++	-	-	0%	
42	41	M	Right	2.6	pT1aN0M0	2	-	+++	-	-	0%	
43	67	M	Left	4.3	pT1bN0M0	2	-	+++	-	-	2%	
44	55	M	Left	5.8	pT1bN0M0	2	-	+++	-	-	1%	
45	73	M	Left	1.6	pT1aN0M0	3	-	+++	-	-	2%	
46	72	M	Right	2.5	pT1aN0M0	2	-	+++	-	-	2%	
47	80	F	Right	2.0	pT1aN0M0	2	-	+++	-	-	4%	
48	49	F	Left	1.5	pT1aN0M0	3	-	+++	+	-	0%	
49	58	M	Left	4.7	pT3aN0M0	2	++	-	ND	-	10%	ND
50	81	F	Left	3.0	pT1aN0M0	2	++	-	ND	+	15%	ND
51	51	M	Left	6.7	pT2aN0M0	3	+	-	ND	-	5%	ND
52	50	M	Right	9.4	pT3aN0M0	3	+	-	ND	-	0%	ND
53	70	F	Right	8.2	pT3aN0M1	4	+	-	ND	-	2%	ND
54	84	F	Left	4.0	pT3aN0M0	3	-	-	-	-	0%	Control
55	56	M	Left	4.0	pT1aN0M0	3	-	-	-	-	3%	Control
56	59	M	Right	3.5	pT3aN0M0	2	-	-	-	-	5%	Control
57	61	M	Left	4.7	pT3aN0M0	3	-	-	-	-	0%	Control
58	63	F	Left	8.1	pT2aN0M0	3	-	-	-	-	0%	Control

TFE3, Cathepsin K IHC: +++ (strong positive), ++ (moderate positive), + (weak expression), - (negative)

*TFE3* wild type, *TFE3* FISH: + (positive), - (negative)

ND: non diagnostic

Control: control group

### Immunohistochemical analysis

**Figure 2:**
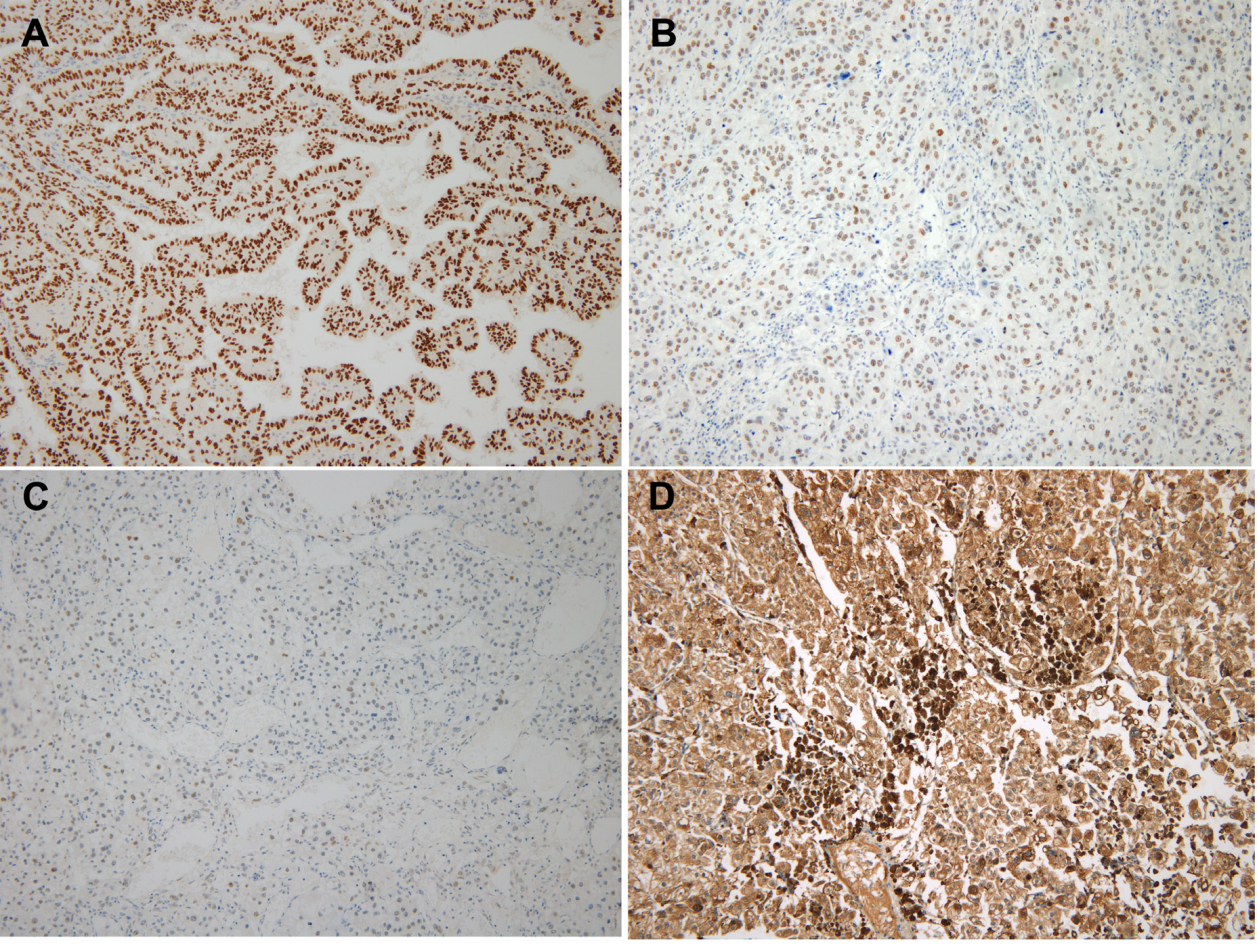
Immunohistochemical staining of TFE3 in Xp11 translocation renal cell carcinoma **A**. TFE3 strong (3+) expression. **B**. TFE3 moderate (2+) expression. **C**. TFE3 weak (1+) expression. **D**. Cathepsin K strong expression.

Nine and seven cases showed strongly and moderately diffuse TFE3 positivity, respectively (Figure 2). Weak expression was detected in 24 cases. Four of the 16 TFE3-positive cases were positive for cathepsin K, which was also observed in three of 24 cases with weak TFE3 expression and 13 of 145 TFE3-negative cases. Overall, 53 cases were more than weakly expressed for TFE3 or positive for cathepsin K. The results are summarized in Table 2.

### FISH analysis

**Figure 3:**
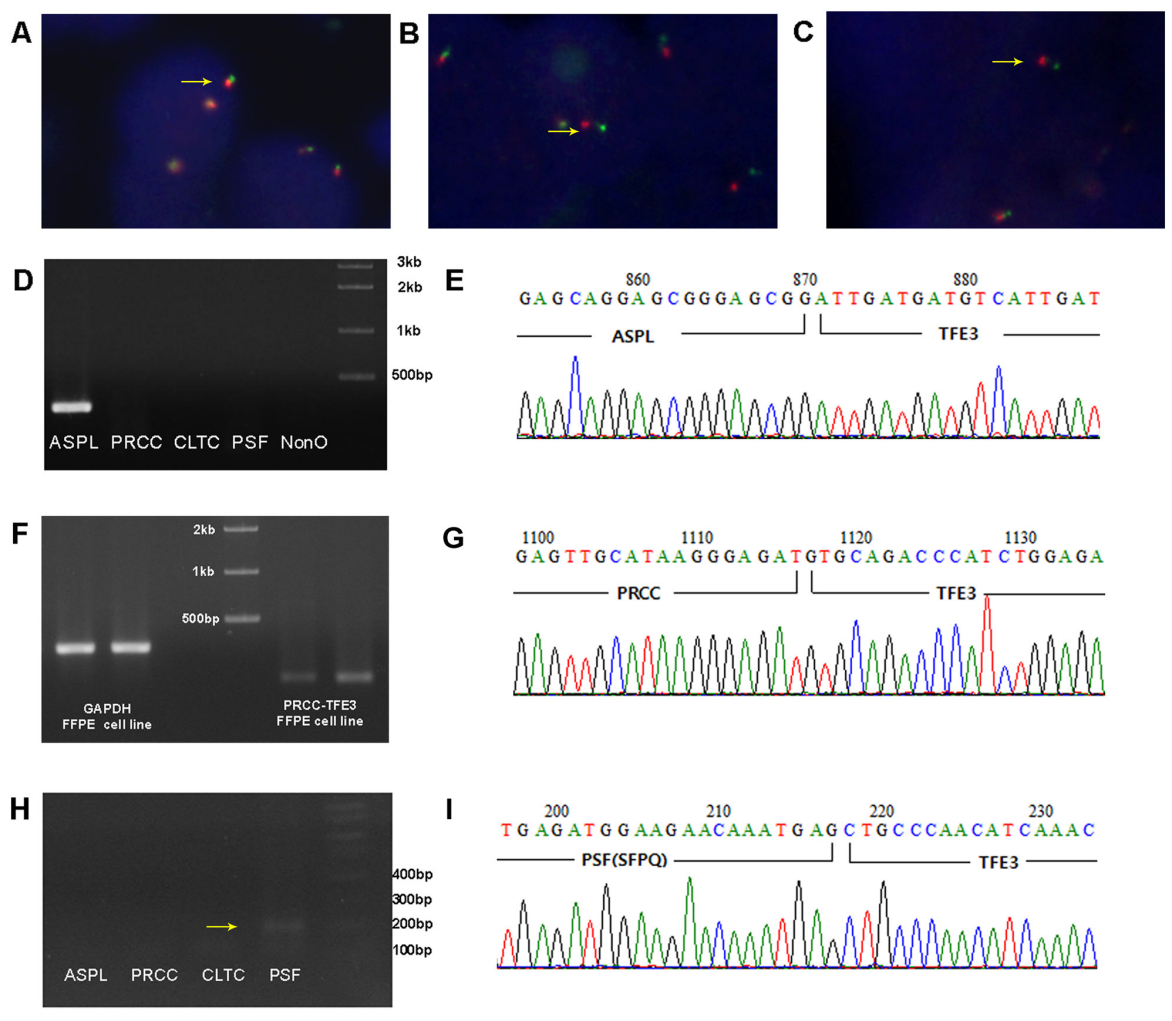
Representative images from the *TFE3* break-apart FISH assay and sequencing analysis of RT-PCR for fusion partners **A**. Normal fusion red-green signals are demonstrated in *TFE3* negative RCC (1000x) **B**. The *TFE3* break-apart probe assay resulted in 1 pair of normal fused hybridization signals and 1 pair of split signals (arrow) in females (1000x). **C**. The *TFE3* break-apart probe assay resulted in 1 pair of split signals (arrow) in males (1000x). **D**. Detection of *ASPL*-*TFE3* translocation by RT-PCR. **E**. Sequencing analysis of *ASPL*-*TFE3* translocation. **F**. Detection of *PRCC*-*TFE3* translocation by RT-PCR of cell line and paraffin tissue. **G**. Sequencing analysis of *PRCC*-*TFE3* translocation. **H**. Detection of *PSF*-*TFE3* tranlocation by RT-PCR with faint solid one band (arrow). **I**. Sequencing analysis of *PSF*-*TFE3* translocation.

We analyzed 53 cases with at least weakly TFE3-positive or with moderate-to-strong cathepsin K expression by FISH and we also tested normal renal tissue with 5 cases of TFE3/Cathepsin K negative tumors for control group (Figure 3A). Of the 16 TFE3-positive cases, 13 (81.3%) were positive by FISH (Table 2). Of the 24 cases with weak TFE3 expression, five were translocation-positive by FISH. None of the 13 TFE3-negative/cathepsin K-positive cases was positive by FISH. Differences in break-apart signal patterns were observed between male and female patients. In the former, positive results consisted of a single pair of separate green and red signals (Figure 3C). In female patients, a positive result was a fused or closely associated green-red signal pair (representing the uninvolved copy of the X chromosome) and an additional pair of split signals (Figure 3B). Samples with single green or red signals were disregarded since they were difficult to accurately interpret.

### RT-PCR for five types of translocation partners

RNA was extracted from 58 cases for RT-PCR analysis; five cases did not meet the exclusion criteria (260/280 and 260/230 ratios > 1.8) and 5 control cases (negative for TFE3 and cathepsin K) were added for RT-PCR. All bands detected in the 25 cases that we suspected having translocations were confirmed by cloning and sequencing: a solid single band at the expected position was confirmed as a translocation. Smearing pattern was revealed to have no translocation by sequencing data, but weak solid one band at proper position was confirmed to have translocation by sequencing analysis (Figure 3H, 3I). *ASPL*- and *PRCC*-*TFE3* translocations were detected in 13/14 (Figure 3D, 3E) and 1/14 (Figure 3F, 3G) TFE3-positive cases, respectively, of which two were excluded. Of 24 cases with weak TFE3 expression, three were dropped, and *ASPL*- and *PRCC*-*TFE3* translocations were demonstrated in three cases each. *PSF*-*TFE3* translocation was observed in one case (Figure 3H, 3I). *ASPL*-*TFE3* translocation was detected in 2/13 TFE3-negative/cathepsin K-positive cases, while two cases harbored *PRCC*-*TFE3* translocation.

### Comparison of immunohistochemical, FISH, and RT-PCR analyses

Relations of immunohistochemistry, FISH and RT-PCR are present in Table 3. All 14 cases showing moderate-to-strong TFE3 expression harbored *TFE3* translocations by RT-PCR, with two cases negative by FISH. *ASPL*- and *PRCC*-*TFE3* fusions were detected in 13 and one case, respectively. There were no cases that were positive by FISH and negative by RT-PCR.

**Table 3:** TFE3 immunohistochemistry with RT-PCR and FISH analysis

Characteristic	Patient cohort					
	TFE3 positive (*n*=14)		TFE3 weak expression (*n*=21)		TFE3 negative, cathepsin K positive (*n*=13)	
	FISH positive	FISH negative	FISH positive	FISH negative	FISH positive	FISH negative
RT-PCR positive	12	2	3	4	0	4
RT-PCR negative	0	0	2	12	0	9

Of the 21 cases with weak TFE3 expression, RT-PCR and FISH results were concordant in three that were positive and 12 that were negative. Four and two cases were positive only by RT-PCR or FISH, respectively. One and three cases harboring *ASPL*- and *PRCC*-*TFE3* fusions, respectively, were negative by FISH. In two samples with weak TFE3 expression that were positive by FISH, no translocation product was detected by RT-PCR. In the weak TFE3 expression group, translocation partners were *ASPL* (*n* = 3), *PRCC* (*n* = 3), and *PSF* (*n* = 1). All 13 TFE3-negative/cathepsin K-positive cases were negative by FISH; however, four showed translocations by RT-PCR (*ASPL*- and *PRCC*-*TFE3*, *n* = 2 each).

### Molecular analysis of cases with weak TFE3 expression

Wild-type (non-translocated) was detected in five cases among 21 TFE3 weak expression cases. This suggests that weak nuclear TFE3 expression is not only a result of translocation, but is also associated with expression of the full-length TFE3 protein. Similar results have been reported in other studies [18, 19].

## DISCUSSION

The diagnosis of translocation RCC has not yet been standardized. An initial diagnosis is usually made based on immunohistochemical detection of TFE3 or TFEB overexpression in the nucleus [13, 20]. However, technical and interpretational challenges remain. For instance, the anti-TFE3 antibody has been shown to be fixation-dependent [13], and it is unclear what intensity of TFE3 immunoreactivity should be considered as positive [21]. Molecular analyses by FISH or RT-PCR are recommended to detect the occurrence of translocation; however, the FISH protocol for *TFE3* has not been fully validated and standardized [12, 21, 22]. Moreover, section truncation signal, which means one signal in male and three signals in female cases, also makes it difficult to interpret correctly [14, 22]. FISH is also a labor-intensive and costly technique. In contrast, RT-PCR has the advantages of being less expensive and able to detect *TFE3* partner genes. Some studies have reported that different types of fusion have distinct prognoses; for instance, *ASPL*-*TFE3* is associated with unfavorable outcome [23, 24]. However, RT-PCR is difficult to implement in clinical pathology laboratories since it is not readily applicable to FFPE tissue [3, 21, 25]. In this study, we used RT-PCR in FFPE specimens, with the results validated by cloning and sequencing to compare the results of immunohistochemistry, FISH, and RT-PCR.

In the 14 cases of moderate or strong TFE3 immunoreactive group, all of these exhibited *TFE3* translocation by either of FISH or RT-PCR. Moreover, immunohistochemistry, FISH, and RT-PCR results were highly concordant, suggesting that moderate or strong TFE3 expression would be indicative of *TFE3* translocation. In the 21 cases of TFE3 weak expressed group, only seven of which harbored *TFE3* translocation. Of the 145 TFE3-negative cases, 13 were cathepsin K-positive; all of these were negative by FISH but four were positive by RT-PCR. It means that when TFE3 immunoreactivity is weak or associated with cathepsin K expression in morphologically suspicious cases, FISH or RT-PCR is recommended to confirm the occurrence of *TFE3* translocation.

Of the 25 cases in which translocation was detected by RT-PCR, 15 were positive by FISH whereas there was no break-apart signal in 10 cases. On the other hand, only two of 17 FISH-positive cases were negative by RT-PCR. This result implies that FISH analysis can yield falsely negative results (10/25, 40.0%) and that RT-PCR would complement FISH analysis for detecting *TFE3* translocation. This is because there may be some ambiguity in interpreting break-apart patterns in FISH. In our experience, the interpretation of the FISH signal was complicated by break-apart signals present in about 5%-15% of tumor cells. The different criterion for calling translocation may be another reason. For example, the distance between break-apart signals to be regarded as positive is one or two signal width depending on the different protocols [15, 21]. Thus, the interpretation of FISH signals is somewhat subjective in borderline cases [26].

RT-PCR cannot be carried out for all FFPE tissue specimens due to inadequate RNA quality. However, only five of 53 cases did not meet the quality criteria in the present study. Reducing the time of warm ischemia after nephrectomy and optimizing the RNA extraction protocol may solve this problem. The interpretation of RT-PCR may also be problematic when the band corresponding to the amplified product is faint; however, in our experience, all faint solid one bands in proper position were found to be associated with translocation upon cloning and sequencing. Another advantage of RT-PCR is that it can identify translocation partners. We detected *ASPL*, *PRCC* and *PSF* for translocation partners. The first two are the most common fusion partners of *TFE3* [2]; thus, three translocation partners can cover more than 90% of *TFE3* partner genes. In our study, the partner genes were 18 for *ASPL*, six for *PRCC*, and one for *PSF*. The cause of this discordance is not clear but relatively small size of cases may be one reason.

The incidence of Xp11 translocation RCC has been variably reported. The largest study examined 443 cases either by cytogenetics or by TFE3 immunohistochemical analysis, and found an incidence of 1.6% [24]. Another group demonstrated that TFE3 was expressed in six of 121 cases (4.9%) [18]. We detected frequencies of 9.7% and 13.5% by FISH and RT-PCR, respectively; this result was confirmed by cloning and sequencing all RT-PCR products. One reason for the lower incidence reported by previous studies may be the fact that immunohistochemistry was used as a primary detection tool, which likely excluded cases with weak expression [13]. In fact, the frequency in the present study was 8.6% when only cases showing moderate to strong TFE3 immunoreactivity were considered. One interesting finding was that most of our patients were not initially diagnosed as translocation RCC, since morphological criteria have yet to be fully defined for this subtype.

In conclusion, we suggest that moderate-to-strong TFE3 staining can be suspicious evidence of Xp11 translocation RCC and the high concordance rate of immunohistochemical and molecular studies is proved. When the protein is weakly or focally expressed, FISH or RT-PCR is recommended to establish a diagnosis for translocation. Cathepsin K expression is non-specific but can be useful for identifying candidates for additional molecular testing. We demonstrated that RT-PCR is highly sensitive and specific and can be used with FFPE tissue. Nonetheless, further studies are necessary to confirm these findings and improve the RT-PCR protocol.

## MATERIALS AND METHODS

### Patient selection

A total of 185 consecutive RCC cases treated at Pusan National University Yangsan Hospital (Yangsan, Korea) between 2011 and 2015 were enrolled in the study. Tissue blocks and accompanying clinical data were collected under a protocol developed by the investigators with Institutional Review Board approval (no. 05-2016-069).

### Immunohistochemistry

All 185 cases were analyzed for TFE3 and cathepsin K expression by immunohistochemistry. FFPE sections (4 μm in thickness) were cut and BOND-MAX autostainer and reagents (Leica BioSystems, Newcastle, UK) were used according to the manufacturer's instructions. Sections were labeled with primary antibodies for TFE3 (MRQ-37; Cell Marque, Rocklin, CA, USA; 1:100) and cathepsin K (3F9; Abcam, Cambridge, MA, USA; 1:1600). TFE3-positive cases were subdivided into moderate (2+) and strong (3+) expression groups based on labeling intensity, according to guidelines of a previous study [13]. Weak immunoreactivity (1+) was defined as subtle nuclear staining at low magnification. Weak (1+) or undetectable (0) TFE3 expression was considered as the TFE3-negative group [13, 27].

### FISH analysis

*TFE3* gene rearrangement was detected in cases showing at weak or higher expression of TFE3 or cathepsin K by dual-color, break-apart FISH using ZytoLight SPEC *TFE3* Dual Color Break Apart Probes (ZytoVision GmbH, Bremerhaven, Germany). Cases positive for *TFE3* by FISH were those expressing split signals in more than 15% of tumor cells [12]. At least 100 nuclei per sample were scored. Only non-overlapping tumor nuclei were evaluated.

### Cell culture

The UOK146 RCC cell line, which harbors the t(X;1)(p11.2;q21.2) translocation that generates chimeric *PRCC*-*TFE3*, was maintained in Dulbecco's Modified Eagle's Medium supplemented with 10% fetal bovine serum and antibiotics (100 U/ml penicillin and 100 μg/ml streptomycin) in a humidified incubator of 5% (v/v) CO_2_.

### RNA extraction and RT-PCR

RNA was extracted from FFPE tissue sections (10 μm in thickness) using an RNeasy FFPE kit (Qiagen, Valencia, CA, USA). RNA of the UOK146 cell was extracted with TRIzol reagent (RiboEx, GeneAll, Seoul, Korea). cDNA was generated using M-MLV reverse transcriptase (Promega, Madison, WI, USA) under the following conditions: 80°C for 3 min; incubation on ice for 5 min; 42°C for 1 h, and 70°C for 15 min. Specimens for which 260/280 or 260/230 nm absorbance ratio was less than 1.8 were excluded due to low RNA quality. PCR reactions were performed with Ex Taq Polymerase (Takara Bio, Otsu, Japan) using the primer set listed in Table 1 under the following conditions: 95°C for 1 min; 35 cycles of 95°C for 20 s, 58°C for 30 s, and 72°C for 27 s; and 72°C for 5 min. Primers were designed to amplify five of the known translocation types (*ASPL*-*TFE3*, *PRCC*-*TFE3*, *PSF*-*TFE3*, *CLTC*-*TFE3*, and *NonO*-*TFE3*) [4] and wild-type (non-translocated) *TFE3* (nucleotides 821-1008).

### Cloning and sequencing

To identify *ASPL*-*TFE3*, *PRCC*-*TFE3*, and *PSF*-*TFE3* transcripts, purified PCR products were ligated into pGEM-T Easy vector (Promega, Madison, WI, USA). The ligation mixture was transformed into competent *Escherichia coli* JM109 cells by heat shock at 42°C. Transformed cells were spread onto agar plates containing ampicillin (100 μg/ml), isopropyl-β-d-thiogalacto-pyranoside (0.5 mM) and 5-bromo-4-chloro-3-indolyl-β-d-galactopyranoside (80 μg/mg). Blue-white screening was carried out to select recombinant plasmids using the Labopass Mini-prep kit (Cosmo Genetech, Daejeon, Korea). These were digested with *Eco*RI, which yielded two bands including the target fragment, which was around 200 bp by 2.0% agarose gel electrophoresis. Fusion transcripts were detected with the M13 20F, and 20R primer combination followed by DNA sequencing using the ABI Big Dye Terminator v.3.1 Cycle sequencing kit (Applied Biosystems, Darmstadt, Germany) on an ABI prism 3730XL sequencer (Applied Biosystems). Sequences were compared by BLAST (National Center for Biotechnology Information, NCBI). Protein homology searches were performed with BLASTX 2.4 (NCBI) and the NCBI Conserved Domain Database v.2.4.
